# Minimal change disease related to rifampicin presenting with acute renal failure during treatment for latent tuberculosis infection: A case report

**DOI:** 10.1097/MD.0000000000010556

**Published:** 2018-06-01

**Authors:** Jee-Seon Kim, Kyong-Ju Kim, Eun-Young Choi

**Affiliations:** a Department of Internal Medicine, Yeungnam University Medical Center; b Department of Pathology, Yeungnam University Medical Center, Daegu, Republic of Korea.

**Keywords:** acute renal failure, dialysis, isoniazid, minimal change disease, rifampicin

## Abstract

**Rationale:**

The standard drugs used to treat tuberculosis are rifampicin and isoniazid. These agents are usually safe and inexpensive for short-term use in treatment of latent tuberculosis infection, but sometimes cause adverse renal effects, including minimal change disease (MCD).

**Patient concerns:**

Here, we report a 51-year-old woman with latent tuberculosis infection who developed nephrotic syndrome during treatment with rifampicin and isoniazid for 25 days.

**Diagnoses:**

Renal biopsy findings were compatible with MCD, and she had no relevant medical history and was not taking other medications. A diagnosis of anti-tuberculosis drug- induced MCD was made. This is the first report of acute renal failure due to rifampicin and/or isoniazid-induced MCD.

**Interventions:**

After cessation of rifampicin and isoniazid, however, acute renal failure progressed and she was treated with temporary dialysis and oral prednisolone.

**Outcomes:**

The patient achieved complete remission after cessation of rifampicin and isoniazid with steroid therapy.

**Lessons:**

This case demonstrates that rifampicin and/or isoniazid can cause nephrotic syndrome with acute renal failure during the first months of continuous latent tuberculosis therapy. Therefore, renal function and proteinuria should be monitored carefully in all patients taking rifampicin and isoniazid, especially during the first few months of therapy.

## 1 Introduction

Rifampicin and isoniazid are the standard drugs used to treat tuberculosis and latent tuberculosis. Rifampicin has been reported to frequently induce adverse renal effects, including 4 cases of minimal change disease (MCD).^[1–4]^ On the other hand, isoniazid can induce severe adverse effects, such as hepatotoxicity, but isoniazid-induced nephrotoxicity has rarely been reported.^[2]^ In addition, only 1 case of isoniazid-induced MCD has been reported.^[5]^ Here, we report a patient with MCD induced by the antituberculosis agents, rifampicin and/or isoniazid. The patient presented with acute renal failure requiring temporary dialysis, and improved after cessation of the drugs with steroid therapy.

## 2 Case report

A 51-year-old woman visited our outpatient clinic because of latent tuberculosis infection detected by a screening examination performed by a healthcare worker. She had no relevant prior medical history. Laboratory findings were normal with a serum creatinine (Cr) level of 0.76 mg/dL (normal 0.6–1.5 mg/dL) and blood urea nitrogen (BUN) level of 12.8 mg/dL (normal 8–23 mg/dL). Antituberculosis treatment was started with isoniazid at 300 mg/d and rifampicin at 600 mg/d. During the 25-day antituberculosis therapy regimen, she complained of nausea, vomiting, general weakness, and edema. Serum Cr and BUN levels were 1.0 and 18 mg/dL, respectively. Rifampicin and isoniazid were discontinued. However, her symptoms progressed for 4 days and urinalysis revealed 4+ proteinuria (normal negative). She was admitted to the hospital for more detailed examinations.

**Figure 1:**
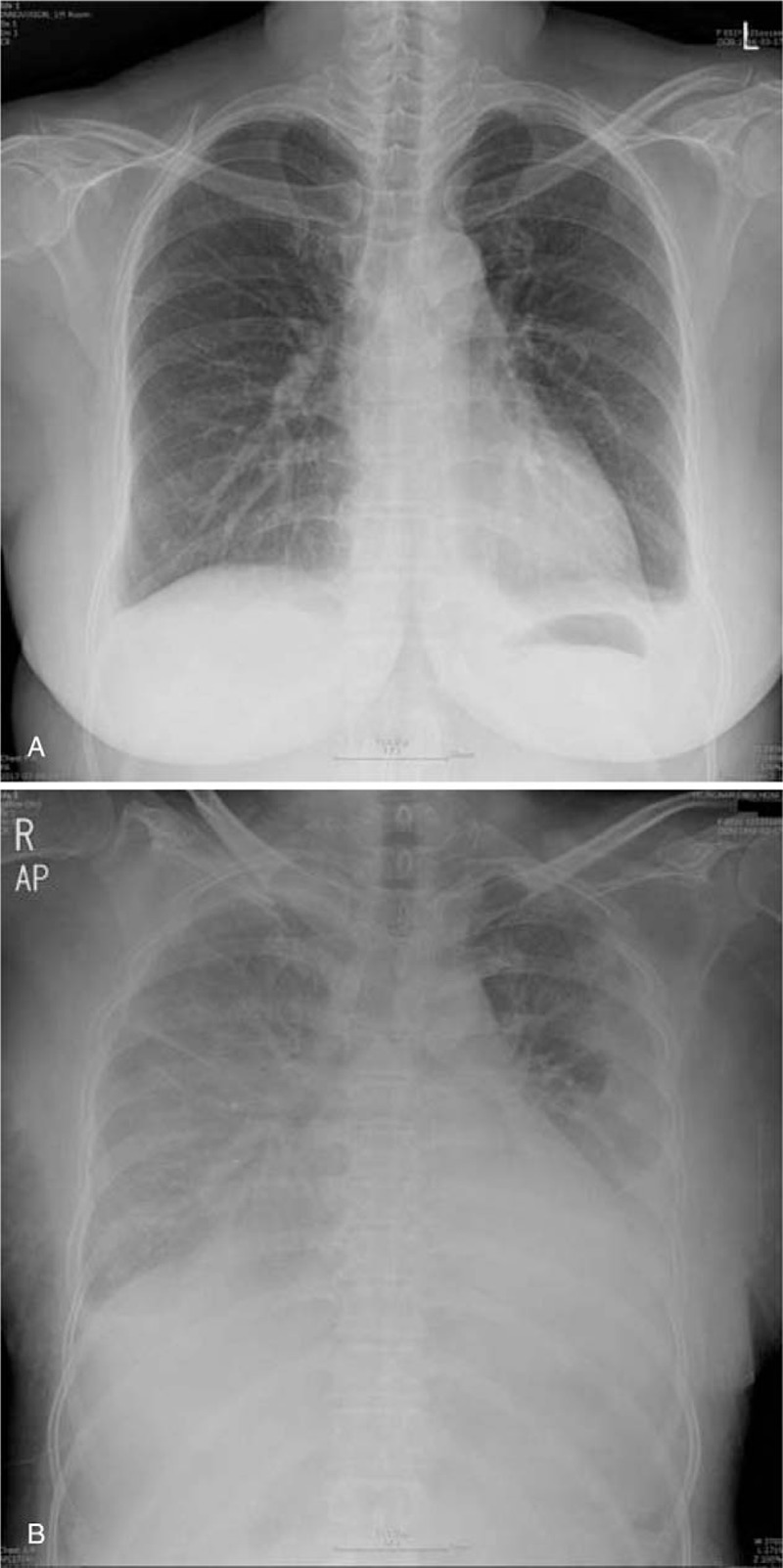
Chest X-ray. (A) On admission, bilateral pleural effusion was detected. (B) Three days later, pulmonary edema developed and pleural effusion was aggravated.

On admission, her blood pressure was 110/80 mm Hg, body temperature was 36.5°C, height was 158 cm, and body weight was 68.6 kg. She had gained 8.6 kg in body weight over the preceding 1 month. The results of physical examination were unremarkable except for pitting edema on both lower extremities. Laboratory findings were as follows: white blood cell count 7490/mm^3^ (normal 4000–10,000/mm^3^) with 63.1% neutrophils and 1.4% eosinophils, hemoglobin 13.6 g/dL (normal 12–16 g/dL), platelet count in peripheral complete blood 295,000/mm^3^ (normal 140,000–440,000/mm^3^), BUN 45 mg/dL, serum Cr 1.72 mg/dL, total protein 3.67 g/dL (normal 6.5–8.2 g/dL), albumin 1.73 g/dL (normal 3.5–5.0 g/dL), total bilirubin 0.67 mg/dL (normal 0.1–1.2 mg/dL), aspartate transaminase 116 IU/L (normal 10–35 IU/L), alanine transaminase 94 IU/L (normal 0–40 IU/L), total cholesterol 453 mg/dL (normal 120–200 mg/dL), sodium (Na) 133 mEq/L (normal 135–145 mEq/L), potassium 5 mEq/L (normal 3.5–5.5 mEq/L), and chloride 103 mEq/L (normal 98–110 mEq/L). Urinalysis showed specific gravity >1.050 (normal 1.005–1.03), osmolality 687 mOsm/kg (normal 300–900 mOsm/kg), urine Na <10 mEq/L, and urinary Cr 267.34. The calculated fractional sodium excretion was 0.02%. The creatinine urine to plasma ratio was 155. Urinary sediment did not show either red blood cells or granular casts. A 24-h urine sample contained 12.2 g of protein. Serum and urine electrophoresis results showed no M-spike and nonselective proteinuria. The patient was negative for hepatitis B, hepatitis C, HIV, and syphilis serological markers. Rheumatoid factor, antinuclear antibody, antineutrophil cytoplasmic antibody, and antiglomerular basement membrane antibody tests were all negative. In addition, results for complement 3 (144.3 mg/dL, normal 90–180 mg/dL), complement 4 (32.4 mg/dL, normal 10–40 mg/dL), immunoglobulin G (551 mg/dL, normal 700–1600 mg/dL), immunoglobulin A (267 mg/dL, normal 70–400 mg/dL), and immunoglobulin M (111 mg/dL, normal 40–230 mg/dL) were negative. Chest X-ray revealed a small amount of bilateral pleural effusion (Fig. 1A). The patient was treated with torsemide at a dose of 50 mg/d for edema.

**Figure 2:**
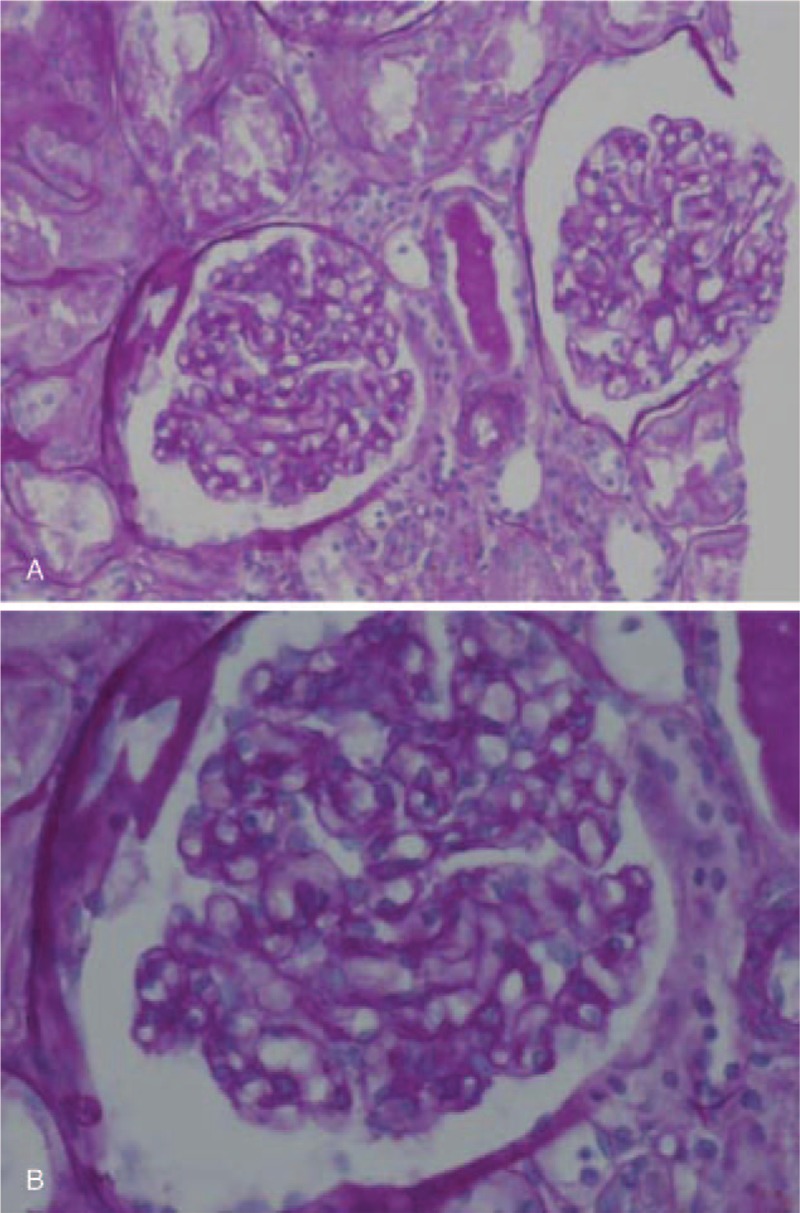
Light micrographs of the renal biopsy specimen. Most glomeruli were normal in terms of size and cellularity. (A) PAS, Periodic acid Schiff, x200 (B) PAS. x400.

**Figure 3:**
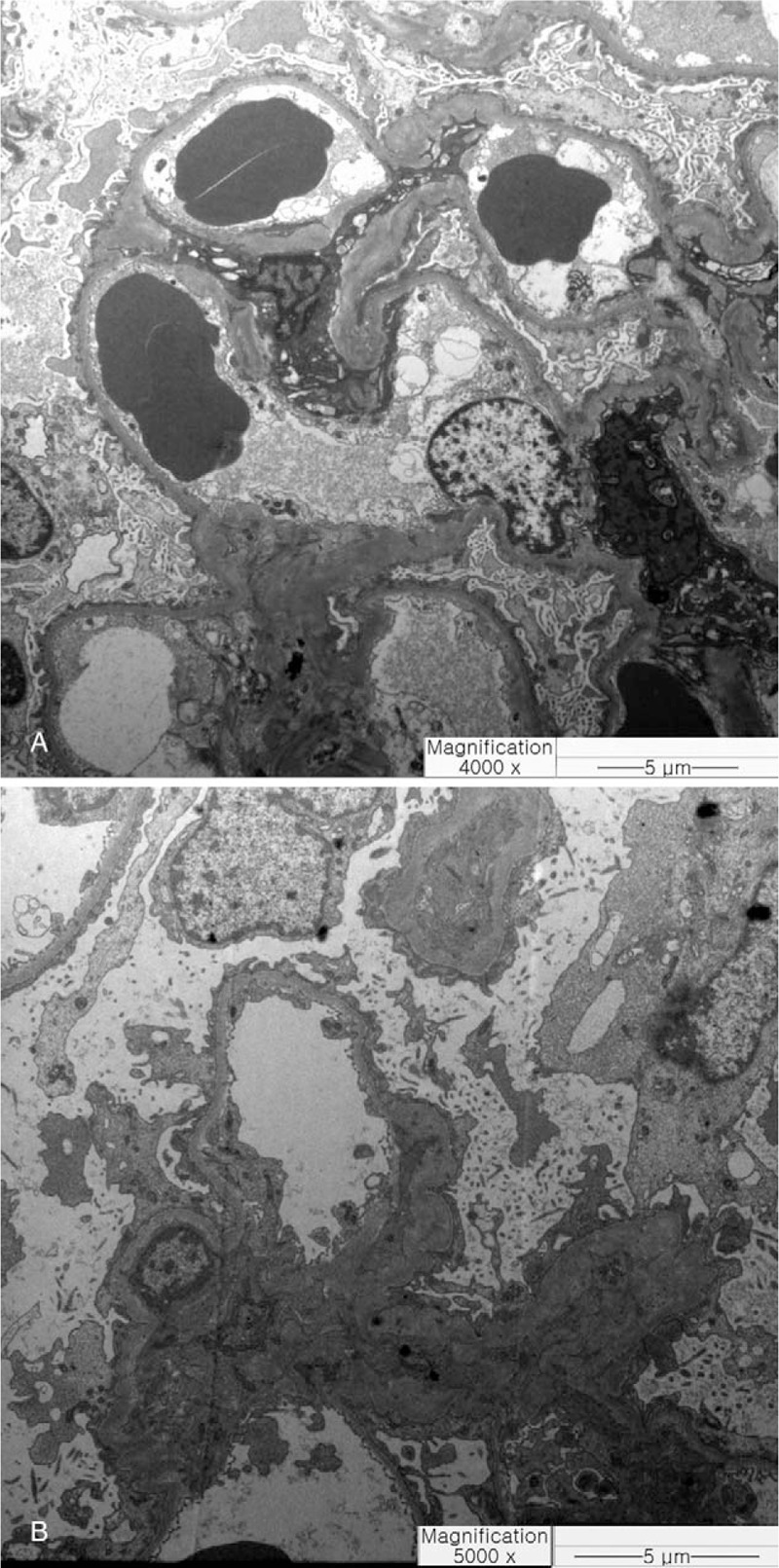
Electron micrographs of the glomeruli. (A) Diffuse loss of the podocyte foot processes of glomerular epithelial cells was seen, and the thickeness and archiecture of the glomerular basement membrane were normal. (B) No electon dense deposits were seen. (Bar = 5 μm).

Renal biopsy was performed at 1 week after discontinuation of medication. However, she developed dyspnea and pulmonary edema on the day of the procedure (Fig. 1B). As we suspected nephrotic syndrome with acute nonoliguric renal failure, we performed dialysis and oral administration of prednisolone at 60 mg/d. Acute renal failure was confirmed with temporary loss of renal function that required dialysis, and with peaked serum Cr (2.68 mg/dL) that more than 3-fold increase in baseline Cr (0.76 mg/dL). Renal biopsy revealed nonsclerotic glomeruli with normocellularity and a mild focal tubular injury pattern on light microscopy (Fig. 2A and B). No deposition of immunoglobulins or complement components was observed in the glomeruli. Electron microscopy showed diffuse loss of the podocyte foot processes of glomerular epithelial cells, but the glomerular basement membrane showed normal thickness and architecture, consistent with MCD (Fig. 3A and B).

**Figure 4:**
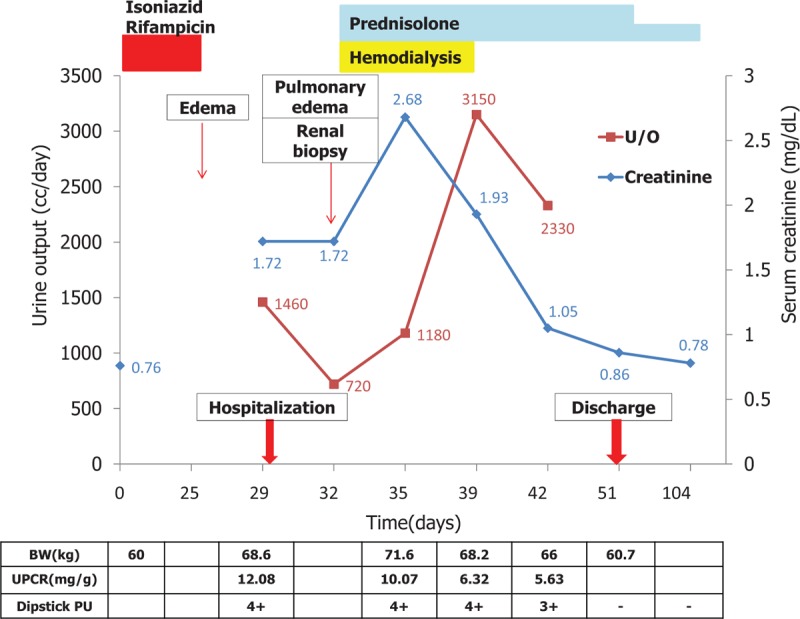
Schematic representation of the patient's clinical course. Systemic edema and nephrotic range proteinuria developed after rifampicin therapy, and progressed to renal dysfunction for 7 days despite discontinuation of rifampicin. The patient's renal function and proteinuria were resolved with the aid of steroid therapy and temporary dialysis.

The diagnosis was confirmed to be MCD. Heavy proteinuria developed after using antituberculosis agents. We speculated that this was a case of antituberculosis medication-induced nephrotic syndrome and toxic hepatitis. Following discontinuation of rifampicin and isoniazid with the aid of prednisolone therapy, the patient's nausea, vomiting, and pulmonary edema improved after 1 week of steroid therapy, and dialysis was stopped. Her body weight recovered from 68.6 to 60.7 kg at 3 weeks after discontinuation of rifampicin and isoniazid, and she was then discharged. Proteinuria became negative and renal function tests showed normal results at 4 weeks (serum Cr, 0.86 mg/dL; BUN, 24.7 mg/dL). Furthermore, her albumin and cholesterol levels were 3.15 g/dL and 276 mg/dL, respectively, at 4 weeks after stopping the drugs. Prednisolone was then tapered and withdrawn 3 months after initiation. Recurrence of proteinuria was not observed during a 3-month follow-up. The patient's clinical course is summarized in Fig. 4.

Informed consents were obtained from the patient for the publication of clinical details and accompanying images. As this study is a clinical case report, no ethical committee approval was required for its conduction, which is in compliance with the institutional and national policies concerning research approvals.

## 3 Discussion

Isoniazid is an effective and inexpensive antituberculosis agent that is commonly used to treat latent tuberculosis infection with a single regimen for 9 months. However, due to its prolonged treatment duration and hepatotoxicity, rifampicin and isoniazid for 3 months is an acceptable alternative treatment regimen for latent tuberculosis infection in real-world practice. Our patient was a nurse with a high likelihood of contact with respiratory tuberculosis patients. Therefore, she was treated with short-term rifampicin and isoniazid for latent tuberculosis infection. During treatment of latent tuberculosis infection for 25 days, the patient developed heavy proteinuria and nephrotic syndrome. In addition, renal biopsy revealed MCD. She had no relevant medical history and was not taking any medications before treatment. Therefore, drug-induced MCD was suspected. Rifampicin occasionally causes adverse renal effects, from mild proteinuria to acute renal failure.^[1–4,6]^ However, isoniazid-induced nephrotoxicity is rare,^[2,5,7]^ and there has been only 1 previous case report of MCD related to this agent.^[5]^ Therefore, physicians usually suspect rifampicin as the causative agent of renal toxicity developing during concurrent therapy with rifampicin and isoniazid.^[1–4]^ In addition, in a previous case series, nephrotic syndrome developed during antituberculosis therapy with rifampicin and isoniazid and resolved after discontinuation of rifampicin; however, proteinuria did not recur despite continuous or interrupted use of isoniazid.^[1–4]^ Therefore, in our case, rifampicin was considered as the most likely causative agent of MCD and acute renal failure, although this was not confirmed by re-administration of isoniazid.

A number of possible mechanisms for rifampicin-induced MCD have been suggested, including a humoral immune mechanism,^[1]^ endothelial injury resulting from rifampicin-induced hemolysis and thrombocytopenia,^[2]^ and direct toxic effects on glomerular epithelial cells.^[4,8]^ Park et al^[4]^ speculated that rifampicin-induced MCD was due to direct toxic effects of the drug, because there was no hemolytic anemia or thrombocytopenia, and no electron-dense deposits or immune complexes in glomeruli to suggest a humoral immune mechanism. As in their case, the mechanism in our case was likely due to direct toxic effects. However, unlike their case, in which resolution occurred spontaneously after cessation of rifampicin, our patient progressed to acute renal failure.

Although there have been a few case reports of antituberculosis agent-induced nephrotic syndrome, acute renal failure due to rifampicin- and/or isoniazid-induced MCD has not been reported previously (Table 1).^[1–6]^ Acute renal failure occurred in 25% to 35% of adults with MCD, and 17% to 20% of these patients required hemodialysis.^[9,10]^ They tended to be older and hypertensive, with lower serum albumin and greater protein excretion than those without acute renal failure.^[9,10]^ Their average serum albumin level was 1.83 to 1.9 g/dL, and proteinuria was 12 to 13 g/d.^[9,10]^ In this case, despite discontinuation of rifampicin and isoniazid, acute renal failure progressed and temporary dialysis was required. The patient's initial blood pressure was 110/80 mm Hg, serum albumin was 1.76 g/dL, and proteinuria was 12 g/dL. Laboratory results, including plasma BUN, Cr, and urinalysis, were compatible with prerenal type acute kidney injury. In addition, there was no evidence of underlying vascular disease or tubulointerstitial lesions on renal biopsy. Plasma volume depletion likely resulted from poor oral intake and excessive diuresis, in addition to a severe nephrotic state resulting in acute renal failure.

**Table 1:**
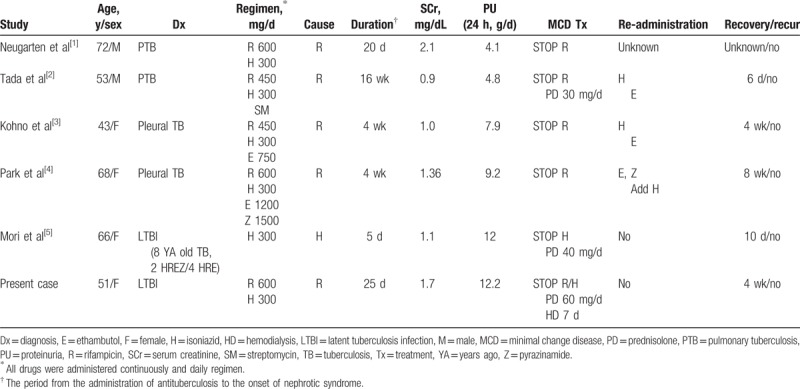
Rifampicin or isoniazid induced MCD reported.

Acute renal failure due to antituberculosis therapy is rare, and usually occurs in patients receiving readministered or intermittent tuberculosis chemotherapy.^[6,11–13]^ Rifampicin-associated acute renal failure with continuous treatment occurs only during the first few months of therapy.^[6,11]^ Isoniazid-induced nephrotoxicity was also reported to occur within 1 month of therapy.^[5,7]^ This patient had not taken rifampicin or isoniazid previously, and developed acute renal failure during the first few months of continuous therapy.

In general, MCD is treated with glucocorticoid, which leads to complete remission in 80% to 95% of adult patients.^[14]^ In previous cases, MCD was improved by cessation of rifampicin and/or isoniazid and oral administration of prednisolone.^[1–3,5]^ Glucocorticoid was not administered in 1 case, and resolved with only discontinuation of rifampicin.^[4]^ However, our patient progressed to acute renal failure despite discontinuation of rifampicin and isoniazid, so we administered oral prednisolone at a dose of 1 mg/kg per day and performed temporary dialysis. She showed improvement of her symptoms, her body weight recovered, and renal function with proteinuria was normalized at 2, 3, and 4 weeks after cessation of the drugs, respectively.

In general, renal prognosis is good in cases of rifampicin- or isoniazid-induced MCD. There have been no reports of mortality among patients, with all patients showing normalization of renal function within several months, and no recurrence of proteinuria in rifampicin- or isoniazid-induced MCD or acute renal failure.^[1–5,11,12,15]^ Our patient recovered full renal function, and we expected her renal prognosis to be good with no additional re-administration of antituberculosis agents.

This is the first case of rifampicin- and/or isoniazid-induced MCD, progressing to acute renal failure requiring temporary dialysis without a fulminant systemic reaction, which improved after cessation of the drugs with steroid therapy.

## 4 Conclusion

Clinicians should be aware of the possibility that rifampicin and/or isoniazid can cause nephrotic syndrome with acute renal failure, even in continuous latent tuberculosis therapy during the first months of treatment. Therefore, all patients taking rifampicin and/or isoniazid should be monitored carefully for renal function and proteinuria, especially in the first months of therapy. Furthermore, in cases of suspected antituberculosis-induced nephrotic syndrome, prompt and accurate diagnosis by renal biopsy and steroid therapy may be necessary in addition to discontinuation of the drug.

## Author contributions

**Resources:** Kyong Ju Kim.

**Writing – original draft:** Jee-Seon Kim.

**Writing – review and editing:** Eun-Young Choi.
